# 405. A Qualitative Study Based on a Case Series of Obstetric COVID-19 Patients to Determine Risks in Management Associated with Severity in a Government Hospital in the Philippines

**DOI:** 10.1093/ofid/ofab466.606

**Published:** 2021-12-04

**Authors:** Sheila Patrice D Funelas-Santos, Judith P Peralta

**Affiliations:** 1 Quirino Memorial Medical Center, Quezon City, National Capital Region, Philippines; 2 Quirino Memorial Medical Center, St. Luke’s Medical Center - Global City, Quezon City, National Capital Region, Philippines

## Abstract

**Background:**

Enterprise Risk Management (ERM) in healthcare is a method used to identify, assess and reduce risk to patients and the hospital organization. The objective of this study is to identify clinical and organizational challenges and risks in healthcare management caused by COVID-19, and its impact on patients and healthcare workers, in a low-resource obstetric setting.

**Methods:**

From a census of patients from 1 April 2020 to 30 July 2020, four cases of COVID-19 in pregnancy representing different severity levels were selected. A patient tracer activity was done for each patient, documenting events that the patient and healthcare team experienced from admission to discharge. A case series on these patients was written. A focus group consisting of an OB-GYN resident, OB-GYN consultant, OB-GYN nurse, OB-GYN infectious disease consultant, and internal medicine resident and consultant, was formed. Each case was presented to the focus group to establish the context of risk assessment. Risks were identified using the framework of Enterprise Risk Management. Each risk was classified according to their risk domain and severity. Root cause analysis via the fishbone method was used to identify the causes of the risks.

**Results:**

Operational risks identified were delayed swab results, false negative swab results, and delayed patient transport. Clinical/Patient risks identified were COVID-19 exposure of healthcare workers and other non-COVID patients, inadvertent community exposure, risk for severe clinical manifestations of COVID-19, and lack of specific treatment for COVID-19. Risk to human capital identified were COVID-19 infection of hospital staff and decreased quantity of workforce due to quarantine. Most risks were assessed to be moderate risk or high risk in terms of severity. Root cause analysis showed that common causes of risks were due to exposure to asymptomatic patients and delayed and false-negative swab results.

**Conclusion:**

The results of this study may be used towards the final steps of ERM: risk evaluation, treatment and management, in a low resource setting.

**Disclosures:**

**Judith P. Peralta,** Doctor of Medicine, Fellow of Philippine Obstetrical and Gynecological Society, Fellow of Philippine Infectious Disease Society for Obstetrics and Gynecology, Research Fellow - Harvard Medical School Department of Obstetrics and Gynecology and Reproductive Medicine (2014-2015), Member - International Society of Infectious Diseases (Brookline, MA), Pfizer (Employee, November 2020 to present)

